# Split Enzyme-Based Biosensors for Structural Characterization of Soluble and Insoluble β-Glucans

**DOI:** 10.3390/ijms22041576

**Published:** 2021-02-04

**Authors:** Daisuke Yamanaka, Suzuka Kurita, Yuka Hanayama, Yoshiyuki Adachi

**Affiliations:** Laboratory for Immunopharmacology of Microbial Products, School of Pharmacy, Tokyo University of Pharmacy and Life Sciences, Tokyo 192-0392, Japan; ymnkd@toyaku.ac.jp (D.Y.); y164092@toyaku.ac.jp (S.K.); yukahanayama5@gmail.com (Y.H.)

**Keywords:** β-1,3-glucan, β-1,6-glucan, β-glucan recognition protein, BGRP, endo-β-1,6-glucanase, luciferase

## Abstract

β-Glucan is widely distributed in various plants and microorganisms and is composed of β-1,3-linked d-glucose units. It may have a branched short or long side chain of glucose units with β-1,6- or β-1,4-linkage. Numerous studies have investigated different β-glucans and revealed their bioactivities. To understand the structure-function relationship of β-glucan, we constructed a split-luciferase complementation assay for the structural analysis of long-chain β-1,6-branched β-1,3-glucan. The N- and C-terminal fragments of luciferase from deep-sea shrimp were fused to insect-derived β-1,3-glucan recognition protein and fungal endo-β-1,6-glucanase (Neg1)-derived β-1,6-glucan recognition protein, respectively. In this approach, two β-glucan recognition proteins bound to β-glucan molecules come into close proximity, resulting in the assembly of the full-length reporter enzyme and induction of transient luciferase activity, indicative of the structure of β-glucan. To test the applicability of this assay, β-glucan and two β-glucan recognition proteins were mixed, resulting in an increase in the luminescence intensity in a β-1,3-glucan with a long polymer of β-1,6-glucan in a dose-dependent manner. This simple test also allows the monitoring of real-time changes in the side chain structure and serves as a convenient method to distinguish between β-1,3-glucan and long-chain β-1,6-branched β-1,3-glucan in various soluble and insoluble β-glucans.

## 1. Introduction

β-Glucans found in yeasts, algae, mushrooms, molds, cereals, bacteria, lichens, and other microorganisms show strong immune-enhancing properties and have been applied to functional food, cosmetics, and animal feed. In particular, soluble β-glucans derived from basidiomycetes have been traditionally used in Japan for cancer treatment. Depending on the origin and purification process, β-glucans vary in molecular weight, solubility, viscosity, and, in particular, the intramolecular side chain conformation. These differences in structure affect their biological activities. For example, yeast cell wall β-1,3-d-glucans are known to have long chain β-1,6-glucosyl branches [1], unlike laminarin [2,3] and schizophyllan (sonifilan, SPG) [4,5]. However, a convenient method to distinguish between these side-chain structural differences, whether soluble or insoluble, has not yet been developed. Therefore, a new method that measures the amount of β-glucan to recognize different structures is essential to promote the development of beneficial β-glucan materials.

We recently constructed both β-1,3-d-glucan- and β-1,6-glucan-specific enzyme-linked immunosorbent assays (ELISAs) using the carbohydrate recognition domain of insect β-1,3-glucan recognition protein (BGRP) and functionally modified endo-β-1,6-glucanase (Neg1-E321Q), respectively [6,7]. *Bombyx mori* (silkworm)-derived BGRP (BmBGRP) showed significant binding to both triple- and single-helical β-1,3-d-glucan [6], and we further artificially modified this BmBGRP to create an improved BGRP (supBGRP) with higher stability. The ELISA system using supBGRP has been successfully applied for quantification of several soluble β-1,3-d-glucan in medicinal mushrooms, and we found that this system is unique in that it does not respond to barley-derived β-1,4-/β-1,3-glucan [8]. Additionally, we generated a new β-1,6-glucan-binding protein (Neg1-E321Q) based on *Neurospora crassa*-derived endo-β-1,6-glucanase (Neg1) to specifically detect β-1,6-glucan structures [7]. As these two β-glucan recognition proteins can be expressed in *Escherichia coli*, they can be fused with various proteins such as *Oplophorus gracilirostris*-derived low molecular weight luciferase [9], NanoLuc. In fact, NanoLuc-fused Neg1-E321Q has helped us to easily detect β-1,6-glucan bound to the membrane in our previous study, and we have found that the units accessible to Neg1-E321Q were β-1,6-linked polymers of glucose with a DP 11–15 or higher [7]. Therefore, Neg1-E321Q can now be used for the specific detection of long-chain polymers of β-1,6-glucan.

In this study, we aimed to verify whether the above two β-glucan binding proteins can be used to detect β-1,3-d-glucan with branches of long-chain β-1,6-glucan polymer at the single-molecule level. We designed proteins using each of the above β-glucan recognition proteins with split-NanoLuc, called NanoLuc Binary Technology (NanoBiT) [10], and constructed a split-luciferase complementation assay for a detailed structural analysis of long β-1,6-branched β-1,3-glucan. The split-luciferase complementation assay has been developed as a tool for monitoring protein–protein interactions [11,12,13] using two proteins that are fused to the two fragments of a reporter enzyme. Accordingly, if the two β-glucan recognition proteins come close together on a single β-glucan molecule, the two fragments of NanoLuc, a large N-terminal (LgBiT) and a small C-terminal (SmBiT) region, recombine and reconstitute full-length NanoLuc that shows enzymatic activity. Here, we establish a new strategy to distinguish β-glucan structures between β-1,3-glucan- and β-1,6-linked glucose polymer branching β-1,3-glucan in a simple operation using split enzyme-fused β-glucan recognition proteins based on the NanoBiT technology.

## 2. Results

### 2.1. Protein Engineering for Development of New Split-NanoLuc Complementation Assay for the Structural Characterization of β-Glucan

**Figure 1:**
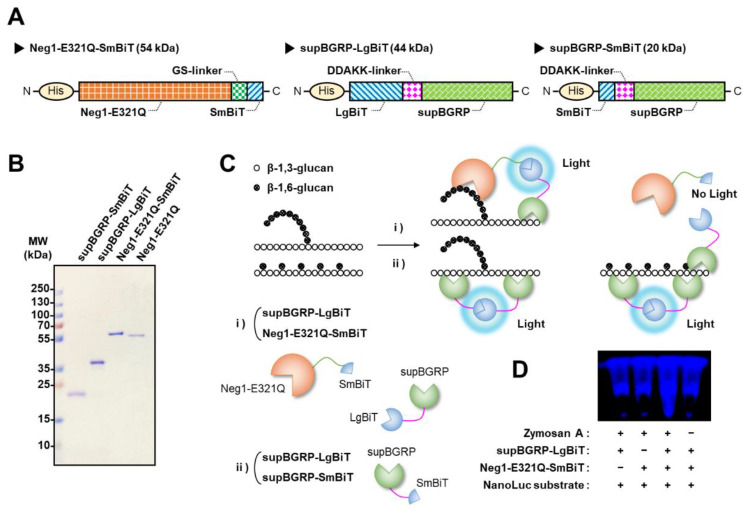
Split-NanoLuc complementation assay for β-glucan. (**A**) Diagram of Neg1-E321Q-SmBiT, supBGRP-LgBiT, and supBGRP-SmBiT. (**B**) SDS-PAGE image of supBGRP-SmBiT, supBGRP-LgBiT, Neg1-E321Q-SmBiT, and Neg1-E321Q. Purified recombinant proteins were separated using a 12% polyacrylamide gel under nonreducing conditions and identified by Coomassie Brilliant Blue staining. (**C**) Schematic illustration of the split-NanoLuc complementation assay for β-glucan. (**D**) Validation of sensor for β-1,6-branched β-1,3-d-glucan by bioluminescence imaging. Zymosan A (50 μg/mL, 40 μL) was mixed with split-NanoLuc-fusion proteins (800 nM each, 10 μL) and furimazine solution (60 μL) in microtubes.

For the analysis of the structural characteristics of various β-glucans without washing, we developed a split-NanoLuc complementation assay using split enzyme-fused β-1,6-glucan- and β-1,3-glucan-specific binding proteins. As most β-glucans are composed of a main chain of β-1,3-glucan, the larger fragment of NanoLuc, LgBiT, was fused to the N-terminal position of supBGRP and the smaller fragment, SmBiT, was fused to the C-terminal side of Neg1-E321Q and the N-terminus of supBGRP with a linker peptide (Figure 1A). These designed proteins could be efficiently expressed in *E. coli* (Figure 1B) and theoretically be used for the unwashed structural analysis of both soluble and insoluble β-glucans. When analyzing β-1,3-glucan, the split-enzyme is reconstituted on the β-1,3-glucan molecule following mixing of supBGRP-LgBiT and supBGRP-SmBiT with the test sample (Figure 1C). When supBGRP-LgBiT was mixed with Neg1-E321Q-SmBiT, the active enzyme could reconstitute only in the presence of β-1,3-glucan structure with a long β-1,6-glucan side chain in the test sample. However, if the side chain comprised a single glucose unit, the reconstitution of NanoLuc would fail owing to the lack of interaction between Neg1-E321Q and short-chain β-1,6-glucan, resulting in no enzyme activity (Figure 1C). To evaluate whether the designed split-NanoLuc fusion β-glucan-binding proteins induced reconstitution of the active form, supBGRP-LgBiT and Neg1-E321Q-SmBiT were mixed with β-glucan particulate and the bioluminescence was monitored by adding the luciferase substrate (furimazine) to the mixture. In this first evaluation, zymosan A, a crude particulate preparation of β-1,3-glucan with highly branched linkages in the 1,6-β-glucan moiety from *Saccharomyces cerevisiae* [14,15,16,17], was used to bring the split-enzyme subunits into close proximity by binding supBGRP and Neg1-E321Q to the surface of the particles. As shown in Figure 1D, bioluminescence was observed only in the presence of supBGRP-LgBiT, Neg1-E321Q-SmBiT, zymosan A, and the substrate. Our results indicate that the split-NanoLuc complementation assay could be used for the screening of several samples for the structural analysis of β-glucan.

### 2.2. Verification of the Reactivity of Split-NanoLuc-Fused Glucan-Binding Proteins to β-Glucans

**Figure 2:**
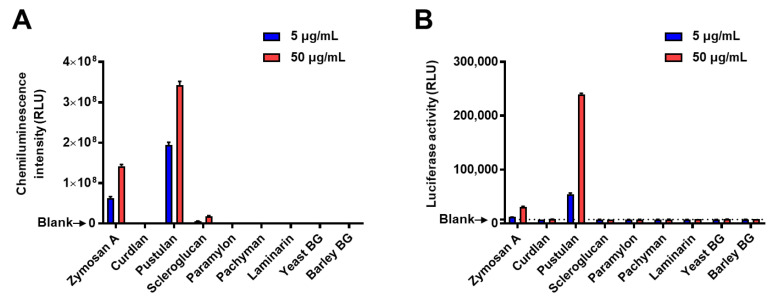
Sandwich ELISA-like assays specific for β-1,6-branched β-1,3-d-glucan using HRP or reconstituted NanoLuc as an indicator. Various glucans (5 or 50 μg/mL) bound to supBGRP-LgBiT immobilized on a microplate were detected using (**A**) biotin-labeled Neg1-E321Q/streptavidin HRP with a chemiluminescence substrate or (**B**) Neg1-E321Q-SmBiT with a luciferase substrate. Data are presented as the mean ± SD of values in duplicates, and are representative results from at least two independent experiments.

We assessed the reactivity of enzyme-fused probes to various β-glucans using 96-well plates coated with β-1,3-glucan probe (supBGRP-LgBiT) following a conventional ELISA-like assay. First, several commercially available β-1,3-glucans (zymosan A, curdlan, pustulan, scleroglucan, paramylon, pachyman, laminarin, yeast BG, and barley BG) were added to the supBGRP-LgBiT-coated ELISA plates, and the bound β-glucan was detected by biotin-Neg1-E321Q or Neg1-E321Q-SmBiT. To detect biotin-Neg1-E321Q, streptavidin-horseradish peroxidase (HRP) and chemiluminescence substrates were used, while reconstituted luciferase activity served as an indicator for the detection of SmBiT-fusion probes. The results showed that zymosan A, pustulan, and scleroglucan were reactive in the sandwich with supBGRP-LgBiT and biotin-Neg1-E321Q (Figure 2A). On the other hand, the sandwich split-enzyme ELISA-like assay using supBGRP-LgBiT and Neg1-E321Q-SmBiT showed reactivity to zymosan A and pustulan, but not scleroglucan (Figure 2B). This difference was likely caused by the higher background of luciferase, which interferes with the detection of weak signals. These results indicate that the β-glucan-binding proteins expressed in *E. coli* maintain their structure-specific binding function even when fused to split-NanoLuc. Furthermore, although the split-NanoLuc was less sensitive than the conventional HRP-based ELISA as a signal indicator, it was demonstrated to be sufficient for use in the structural analysis.

### 2.3. Development of the Split-NanoLuc Complementation Assay for β-Glucan

**Figure 3:**
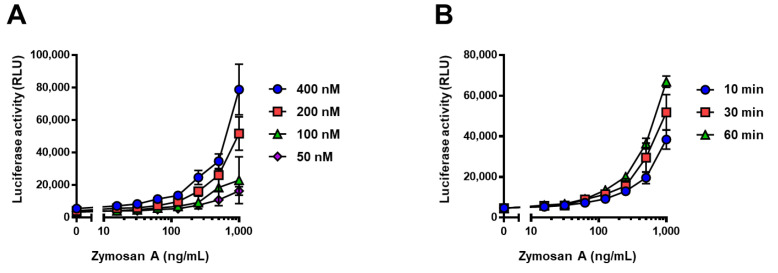
Effect of different concentrations and incubation time periods on split-NanoLuc complementation assay. (**A**) Different concentrations of mixture of supBGRP-LgBiT and Neg1-E321Q-SmBiT (5 μL) were incubated with 10 μL of zymosan A (0–1000 ng/mL) for 30 min. (**B**) Zymosan A (0–1000 ng/mL, 10 μL) was incubated with the mixture solution of supBGRP-LgBiT and Neg1-E321Q-SmBiT (200 nM each, 5 μL) for different time points (10, 30, and 60 min). After incubation, 15 μL of furimazine solution were added and NanoLuc activity was measured. Data are presented as the mean ± SD of values in four experiments.

To construct and optimize the no-wash split-NanoLuc complementation assay, the reactivity was investigated at different concentrations of glucan probes and different reaction times. Different concentrations of the mixture solution of supBGRP-LgBiT and Neg1-E321Q-SmBiT were incubated with zymosan A in a 96-well plate for 30 min to assess the reconstructed enzyme activity. As shown in Figure 3A, the enzyme activity increased in proportion to the concentration of each probe. A suitable reaction curve was obtained in the presence of at least 200 nM of each probe. Next, 200 nM mixture solution of supBGRP-LgBiT and Neg1-E321Q-SmBiT was incubated in a 96-well plate with zymosan A for 10, 30, and 60 min, and the enzyme activity was measured. The results showed that 10 min of incubation was sufficient to obtain a reaction curve; nevertheless, further incubation resulted in an increase in enzyme activity (Figure 3B). On the basis of these results and the consumption of protein probes, we decided to use the method with 200 nM of probes and 30 min incubation for assessing the reconstructed enzyme activity in subsequent studies.

**Figure 4:**
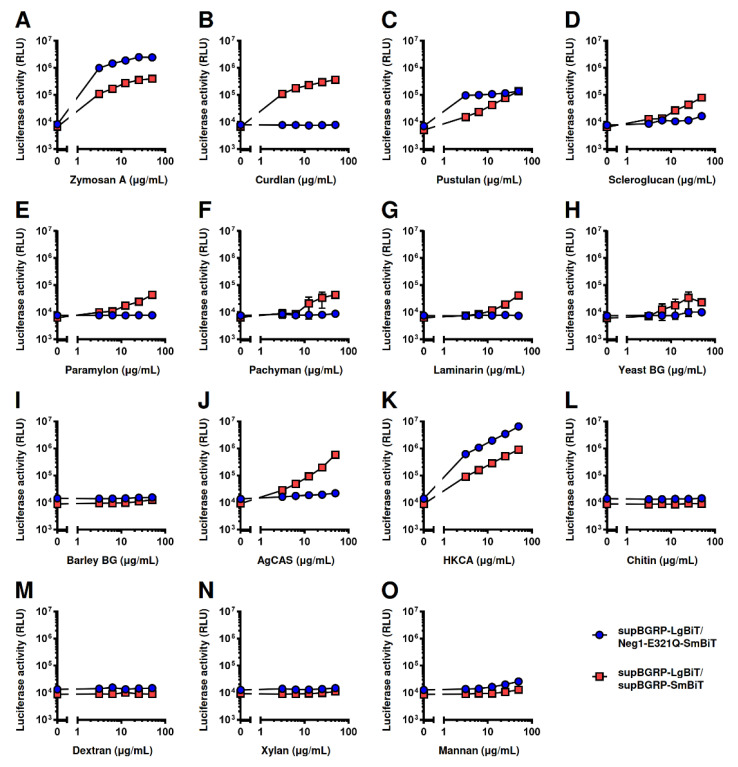
Measurement of various glucan samples using split-NanoLuc complementation assay. The mixture solution of supBGRP-LgBiT and Neg1-E321Q-SmBiT (200 nM each, 5 μL) for β-1,6-branched β-1,3-d-glucan or supBGRP-LgBiT and supBGRP-SmBiT (200 nM each, 5 μL) for β-1,3-d-glucan was incubated with test samples, (**A**) zymosan A, (**B**) curdlan, (**C**) pustulan, (**D**) scleroglucan, (**E**) paramylon, (**F**) pachyman, (**G**) laminarin, (**H**) yeast BG, (**I**) barley BG, (**J**) AgCAS, (**K**) HKCA, (**L**) chitin, (**M**) dextran, (**N**) xylan, and (**O**) mannan (0–50 μg/mL each). After 30 min, 15 μL of furimazine solution were added and reconstituted NanoLuc activity measured. Data are presented as the mean ± SD of values in duplicates and are representative results from at least two independent experiments.

Next, we evaluated the reactivity of the split-NanoLuc complementation assay with various polysaccharides (Table 1). The combination of supBGRP-LgBiT and supBGRP-SmBiT showed strong reactivity to zymosan A, curdlan, and pustulan (Figure 4A–C) and weak reactivity to scleroglucan, paramylon, pachyman, laminarin, and yeast BG (Figure 4D–H). However, this combination failed to respond to barley BG, a β-1,4-/β-1,3-glucan (Figure 4I). In addition, the β-1,3-glucan structure showed reactivity to crude samples, including mushroom-derived AgCAS and heat-killed *Candida albicans* (HKCA) (Figure 4J,K). Other polysaccharides such as chitin, dextran, xylan, and mannan without β-1,3-glucan structure (Figure 4L–O) showed no reactivity. In contrast, the combination of supBGRP-LgBiT and Neg1-E321Q-SmBiT exhibited a strong response to long β-1,6-branched-β-1,3-glucans such as zymosan A, pustulan, and HKCA (Figure 4A,C,K) and weak reactivity toward scleroglucan and yeast BG (Figure 4D,H). These results demonstrate that the split-NanoLuc complementation assay constructed herein can be applied for the detection of β-1,3-glucan or long β-1,6-branched-β-1,3-glucan.

### 2.4. Application for the Analysis of the Composition and Structure of the Fungal Intact Cell Wall

**Figure 5:**
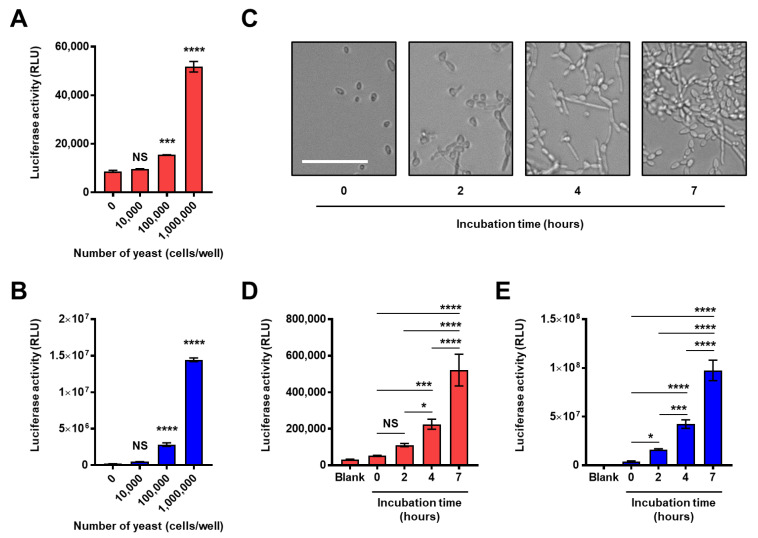
Reconstitution of NanoLuc on the cell surface of live *C. albicans*. (**A**,**B**) Reactivity to yeast form *C. albicans*. Yeasts in 96-well white V-bottom plate (0, 10^4^, 10^5^ or 10^6^ cells/well) were washed, treated with the mixture solution of (**A**) supBGRP-LgBiT and supBGRP-SmBiT or (B) supBGRP-LgBiT and Neg1-E321Q-SmBiT, and the NanoLuc activity measured. (**C**) Images showing the proliferation of *C. albicans* (10^5^ cells/well) in a 96-well clear flat bottom plate. (**D**,**E**) Reactivity to hypha form *C. albicans*. Yeasts in 96-well white V-bottom plate (10^5^ cells/well) were cultured for different time periods (0, 2, 4 or 7 h) at 37 °C. The hyphae were washed and analyzed with (**D**) supBGRP-LgBiT and supBGRP-SmBiT or (**E**) supBGRP-LgBiT and Neg1-E321Q-SmBiT. Data are presented as the mean ± SD (*n* = 3 for A, B, *n* = 4 for D, E) and are representative results from at least two independent experiments. Significant differences from blank (Tukey’s test for A, B) or between different incubation time (Dunnett’s test for D, E): * *p* < 0.05, *** *p* < 0.001, **** *p* < 0.0001, NS: not significant. Scale bar: 50 μm.

Next, we expanded our investigation into the structural analysis of the natural cell wall of viable fungi. Our experiment demonstrated the ability of the split-NanoLuc complementation assay to determine the presence of β-1,3-glucan or long β-1,6-branched-β-1,3-glucan on the surface of HKCA (Figure 4K), however, HKCA does not mimic the natural cell wall composition of *C. albicans* because heat treatment often removes the mannan layer masking the surface of β-glucan [18]. Therefore, we tested whether this assay could be applied to the analysis of natural cell wall structures in living fungi. First, different numbers of the yeast form *C. albicans* were washed and mixed with the split-NanoLuc-fused β-glucan binding protein sets in phosphate-buffered saline (PBS). Then, reconstituted NanoLuc on the viable cell surface was measured, and our results showed a significant increase in luciferase activity in both the constructs of supBGRP-LgBiT/supBGRP-SmBiT and supBGRP-LgBiT/Neg1-E321Q-SmBiT in response to more than 10^5^ yeast cells (Figure 5A,B). Second, we assessed the potential of this assay for the direct analysis of cell wall glucan structures of the hyphal form *C. albicans*. To avoid live *C. albicans* and the reporter proteins interfering with each other, *Candida* cells were cultured independently for different hours in a white V-bottom plate, and washed to remove soluble glucans. The microscopic morphology of *C. albicans* cultured under similar conditions in parallel using clear flat plates at each incubation time is shown in Figure 5C. The split-NanoLuc-complementation assay was carried out on gently-washed viable hyphae in a V-bottom plate, and the activities of luciferase, indicating the amount of surface β-1,3-glucan or long β-1,6-branched-β-1,3-glucan significantly increased in a growth-dependent manner (Figure 5D,E). These results support the utility of this assay for the analysis of glucan structures of intact fungal cell walls.

### 2.5. Application of the Split-NanoLuc Complementation Assay for Real-Time Monitoring of Structural Changes in β-Glucan

**Figure 6:**
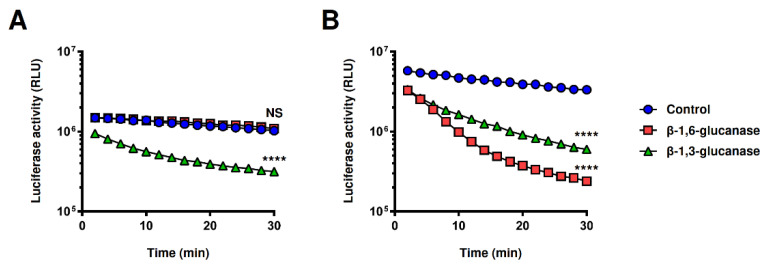
Real-time monitoring of side-chain structural changes of β-glucan using split-NanoLuc complementation assay. The mixture solution of (**A**) supBGRP-LgBiT and supBGRP-SmBiT or (**B**) supBGRP-LgBiT and Neg1-E321Q-SmBiT (200 nM each, 5 μL) were incubated with 10 μL of zymosan A (10 μg/mL) for 60 min. After incubation, endo-β-1,6-glucanase or endo-β-1,3-glucanase (0.5 μg/mL, final) was added with 15 μL of furimazine solution, and reconstituted NanoLuc activity was measured 15 times every 2 min. Data are presented as the mean ± SD (*n* = 4) and are representative results from at least two independent experiments. Significant differences relative to untreated (control) sample at 30 min (unpaired *t*-test) are represented as **** *p* < 0.0001; NS: not significant.

The split-NanoLuc complementation assay, in theory, can monitor the time-dependent structural changes in ligands in real time, unlike conventional ELISA using HRP. For instance, the structure of the cell wall glucan is dynamically changed following the action of various enzymes during fungal growth. Therefore, we treated zymosan A with endo-β-1,6-glucanase (Neg1) or endo-β-1,3-glucanase (Zymolyase) and examined whether the time-dependent degradation of the structure of β-1,6-linked β-glucan side chain or β-1,3-glucan chain could be evaluated by the split-NanoLuc complementation assay. The zymosan A suspension was pre-incubated with the supBGRP-LgBiT/supBGRP-SmBiT or supBGRP-LgBiT/Neg1-E321Q-SmBiT, and luciferase substrates were mixed and then treated with PBS, endo-β-1,6-glucanase, or endo-β-1,3-glucanase. The reconstituted enzyme activity was measured for 30 min. As shown in Figure 6A, the luminescence intensity gradually decreased for untreated control samples (spontaneous decay). In contrast, the endo-β-1,3-glucanase-treated zymosan A sample showed a rapid decrease in luminescence; the measured RLU value after 30 min was approximately one third of that the untreated group. Interestingly, the degradation of the β-1,6-linked side chain on zymosan A did not affect the glucan-binding capacity of supBGRP. Conversely, the activity of the reconstituted NanoLuc from the combination of supBGRP-LgBiT and Neg1-E321Q-SmBiT was dramatically reduced by treatment with endo-β-1,6-glucanase or endo-β-1,3-glucanase (Figure 6B). The measured RLU values after 30 min were reduced to approximately 1/6th and 1/14th for the endo-β-1,3-glucanase and endo-β-1,6-glucanase-treated groups, respectively, compared to the untreated group. These results suggest that the split-NanoLuc complementation assay is not only suitable for the structural analysis of β-glucan but also for real-time monitoring of changes in the structures of long β-1,6-branched-β-1,3-glucans.

### 2.6. Comparison of Reactivity between Conventional HRP-Based ELISA and Split-NanoLuc Complementation Assay for Detection of Soluble and Insoluble β-Glucan

**Figure 7:**
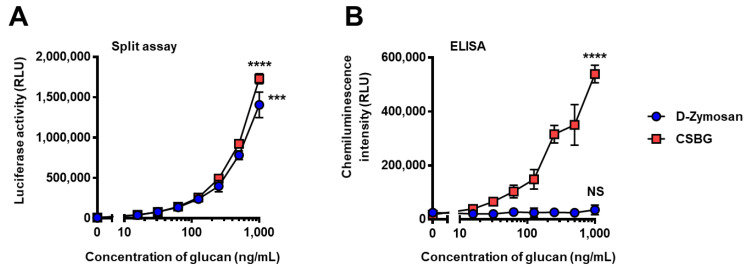
Comparison of split-NanoLuc assay and ELISA for detecting soluble and insoluble β-glucans. (**A**) Response to the split-NanoLuc complementation assay. The mixture solution of supBGRP-LgBiT and Neg1-E321Q-SmBiT (200 nM each, 5 μL) was incubated with 10 μL of depleted zymosan (D-Zymosan) suspension or CSBG solution (0–1000 ng/mL, each). After 30 min, 15 μL of furimazine solution were added and NanoLuc activity measured. (**B**) Response to the HRP-based ELISA method. D-Zymosan suspension and CSBG solution (0–1000 ng/mL) bound to a plate coated with supBGRP-LgBiT were detected using Neg1-E321Q-biotin and streptavidin-HRP with a chemiluminescence substrate. Data are presented as the mean ± SD (*n* = 4) and are representative results from at least two independent experiments. Significant differences between blank and the highest concentration (1000 ng/mL) are indicated as *** *p* < 0.001; **** *p* < 0.0001; NS: not significant.

β-Glucan exists in a particulate or soluble form depending on its origin and purification method. Although soluble β-glucan can be easily detected by conventional HRP-based ELISA, the accurate measurement of insoluble β-glucan is rather difficult. To understand the performance of the split-NanoLuc complementation assay, its reactivity to soluble or insoluble β-glucan was compared with that of the conventional HRP-based ELISA. A hot alkali-treated zymosan (depleted zymosan) was washed multiple times to remove the water-soluble fraction, and the suspension of depleted zymosan was used as the insoluble long β-1,6-branched β-1,3-glucan. An aqueous solution of *Candida* soluble β-glucan (CSBG) purified from the *Candida* cell wall served as a soluble long β-1,6-branched β-1,3-glucan (Table 1). The mixture solution of supBGRP-LgBiT and Neg1-E321Q-SmBiT was further incubated with soluble or particulate β-glucan in a microplate under shaking conditions for 30 min, and bioluminescence was measured after the addition of the luciferase substrate. The results showed a dose-dependent increase in β-glucan reactivity toward both soluble and insoluble forms (Figure 7A). The HRP-based sandwich ELISA method showed a dose-dependent increase in reactivity in soluble CSBG but not in insoluble depleted zymosan (Figure 7B). These results demonstrate that the split-NanoLuc complementation assay provides a technique for the rapid, primary structure-specific analysis of both soluble and insoluble β-glucans in different samples under the same conditions.

## 3. Discussion

Analytical methods such as nuclear magnetic resonance (NMR) and chromatography have been used to investigate the detailed structure of β-glucan [19] but often require highly purified and solubilized samples prepared through multiple procedures. Several biochemical methods have been developed to measure β-1,3-glucan, including the *limulus* amebocyte lysate (LAL) assay and LAL-related methods using the β-1,3-d-glucan recognition molecule (factor G) in horseshoe crabs [20,21,22] as well as those using the β-1,3-glucan recognition proteins in mammals, such as antibodies and dectin-1 (the primary receptor for β-1,3-glucan) [23,24,25]. In addition, to quantify β-1,6-branched β-1,3-glucan, hetero-sandwich ELISA and the glucan enzymatic method (GEM assay) combining chemical and enzymatic hydrolysis, such as α-glucanase and β-glucanase, have been developed [26,27,28]. However, ELISA is generally unsuitable for the analysis of water-insoluble ligands, and the GEM assay and many related methods have not been designed to directly detect β-1,6-branched β-1,3-glucan at the molecular level.

In the present study, we developed no-wash convenient assays to detect the amount of long β-1,6-branched β-1,3-glucan or β-1,3-glucan in test samples. Note that in the case of combining supBGRP-LgBiT and Neg1-E321Q-SmBiT, the NanoLuc activity is affected by the frequency of long β-1,6-branching and the balance between the β-1,3-glucan and β-1,6-glucan, as they target different structures. The difference between the activity of the reporter protein observed in the absence of the target β-glucan (background) and the reconstituted reporter protein in the presence of β-glucan was approximately 100-fold (e.g., zymosan A). Interestingly, the structure of water-insoluble β-glucan, which is underestimated or not optimally evaluated by HRP-based ELISA, could be analyzed under conditions similar to those for soluble β-glucans. Furthermore, this method could be adapted to analyze the cell wall composition of live fungi. This would be the advantage of this detection system. Therefore, the application of this rapid assay allows high-throughput screening of the structure of β-glucan in a large number of test samples and accelerate the development of new functional foods, bio-related materials, and so on.

We have demonstrated our strategy using the original two glucan-binding proteins; however, there are still some limitations to this method, due to the functions of probes. For example, the reactivity of supBGRP to β-1,3-glucan was variable. We evaluated the elemental composition of several commercial β-1,3-glucans and confirmed most of them, except for zymosan A, scleroglucan, and yeast BG, had very low nitrogen and normal carbon content (Table 1). Therefore, the difference in reactivity between supBGRP and each β-1,3-glucan could not be attributed to differences in the purity of the samples, but rather to simple differences in the binding properties of supBGRP, which may depend on the branching frequency and molecular weight. However, since the enzymatic degradation of β-1,6-glucan side chains did not significantly affect the binding of supBGRP and there was no reactivity with a barley-derived β-1,4-/β-1,3-glucan, the ratio and molecular size of the β-1,3-glucan moiety in the molecule may have a greater impact on the binding. Notably, because BGRP is a key molecule that triggers the host defense system in insects, β-glucans that strongly activate the innate immune system are highly reactive to supBGRP. In addition, due to the unique ability of Neg1-E321Q to recognize long-chain β-1,6-glucan, it is not possible to distinguish between unbranched β-glucan (e.g., curdlan) and short-chain branched β-glucan (e.g., laminarin). To overcome this limitation, new probes for short-chain β-1,6-glucan will need to be developed and combined in the future.

The immunomodulatory effects of β-1,3-glucan have been evident after the discovery of the major β-1,3-glucan receptor, dectin-1, in the early 2000s [29,30]. However, how the minimum structural differences in β-glucans affect their biological activities is questionable. The immunomodulatory effects of β-1,3-glucan are known to be affected by differences in the solubility, frequency, and length of branching as well as molecular weight [31,32,33,34]. As it was not easy to carry out analysis and comparison of subtle differences in the structures of β-1,3-glucans used in each study, only limited information about detailed β-glucan structures has been reported. Future studies should perform a detailed structural analysis of glucan to understand the relationship between the structural diversity of β-glucan and changes in biological activities.

## 4. Materials and Methods 

### 4.1. Materials

Laminarin (soluble β-1,3-glucan polymer) from *Laminaria digitate*, β-d-glucan from barley (barley BG), zymosan A and mannan (α-1,6-/α-1,2-, α-1,3-mannan) from *S. cerevisiae*, and bovine serum albumin (BSA) were purchased from Sigma-Aldrich (St. Louis, MO, USA). Dulbecco’s PBS, curdlan, paramylon, chitin, and dextran were obtained from Wako Pure Chemical Industries, Ltd. (Osaka, Japan). Pustulan (soluble β-1,6-glucan polymer) from *Lasallia pustulata* and hot alkaline-treated zymosan (depleted zymosan) were purchased from InvivoGen (San Diego, CA, USA). Scleroglucan was purchased from CarboMer, Inc. (San Diego, CA, USA). Pachyman was procured from Calbiochem (San Diego, CA, USA), and yeast β-1,3-/β-1,6-glucan (yeast BG) and corn core xylan were obtained from Megazyme (Bray, Ireland) and Tokyo Chemical Industry Co., Ltd. (Tokyo, Japan), respectively. Streptavidin-conjugated HRP was provided by R&D Systems (Minneapolis, MN, USA), and Coomassie Brilliant Blue (Rapid CBB KANTO, CBB-R250) by Kanto Kagaku Co. (Tokyo, Japan). The endo-β-1,3-glucanase (zymolyase 100T) was purchased from Seikagaku Corp. (Tokyo, Japan). Outdoor-cultivated fruiting bodies of *Agaricus brasiliensis* strain KA21 were provided by Toei Shinyaku Co., Ltd. (Tokyo, Japan). *C. albicans* NBRC 1385 and *N. crassa* NBRC 6068 were obtained from the NITE Biological Resource Center (Chiba, Japan). The polysaccharide samples used in this study are listed in Table 1. The elemental composition of several polysaccharide products was confirmed by elemental analysis using an Elementar Vario EL Cube analyzer (Elementar Analysensysteme GmbH, Langenselbold, Germany).

### 4.2. Preparation of Mushroom-Derived Polysaccharide Fraction

The cold alkaline-soluble fraction of the fruiting body of *A. brasiliensis* (AgCAS) extracted with sodium hydroxide solution after repeated extraction with water was prepared according to a previous report and used as the β-1,6-glucan-rich water-soluble fraction [35].

### 4.3. Preparation of HKCA and Cell-Wall Solubilized β-Glucan

*C. albicans* was seeded in sterile yeast extract, peptone, and dextrose (YPD) liquid medium (200 mL) in a Sakaguchi flask and cultured at 27 °C for 48 h. The yeast form of *C. albicans* was treated by autoclaving. After centrifugation, cells were washed twice with deionized water and finally mixed with 250 mL water and stirred for 1 day at 4 °C. The extracted components were removed by centrifugation. Cells were washed twice with ethanol, dried by acetone treatment, resuspended in water, and used as HKCA. Purified solubilized long-chain β-1,6-glucan branching β-1,3-glucan CSBG from *C. albicans* NBRC 1385 was prepared according to a previous report [36].

### 4.4. Plasmid Construction

The mature form of recombinant endo-β-1,6-glucanase (EC 3.2.1.75), Neg1, classified into glycoside hydrolase (GH) family 30 in the Carbohydrate-Active enZymes database (CAZy; www.cazy.org), and its derivative, β-1,6-glucan-specific probe Neg1-E321Q, was prepared as previously reported [7,37]. Briefly, the β-1,6-glucanase-coding gene (*neg1*) was amplified by PCR using PrimeSTAR Max DNA Polymerase (Takara Bio Inc., Shiga, Japan) from *N. crassa*-derived cDNA. The PCR amplicons were cloned into a linearized pCold I DNA vector (Takara Bio Inc.) (1–300, 361–4407) using an In-Fusion HD Cloning Kit (Clontech Laboratories, Inc., CA, USA) (pCold-Neg1). The point mutation at the catalytic domain [38] of Neg1-Glu^321^ (nucleophile) to Gln was induced to prepare a β-1,6-glucan-specific probe (pCold-Neg1-E321Q). The small C-terminal region (SmBiT) of *O. gracilirostris*-derived NanoLuc was inserted into pCold-Neg1-E321Q at the C-terminus of Neg1-E321Q. The linear vector encoding Neg1-E321Q without a stop codon was amplified using specific primer sets (pColdI-n361-F and NEG1-FS-R). The DNA fragment encoding SmBiT was combined across the flexible GS linker peptide, Gly-Gly-Ser-Gly-Gly-Gly-Ser-Gly-Gly (GGSGGGSGG) sequence. The sequences of the synthetic DNA fragments used in this study are listed in Table 2. The β-1,3-glucan-specific probe, supBGRP, and the expressing plasmid vector were designed and prepared with reference to the previous report [6]. The linear vector encoding supBGRP was amplified and the small C-terminal region (SmBiT) or large N-Terminal region (LgBiT) of NanoLuc was inserted into the pCold vector encoding supBGRP at the N-terminus of supBGRP. A flexible DDAKK linker with four repeats of Asp-Asp-Ala-Lys-Lys (4DDAKK) was inserted between supBGRP and split NanoLuc. All protein-expressing plasmid vectors were purified from *E. coli* DH5α competent cells, which were cultured in Luria Bertani (LB) broth containing ampicillin (100 μg/mL). The DNA sequences were confirmed before use.

### 4.5. Preparation of Split Enzyme-Fused Glucan-Binding Proteins

Expression vectors for Neg1- and supBGRP-derivatives were transformed into SHuffle express competent *E. coli* (New England Biolabs Inc., Ipswich, MA, USA) and *E. coli* BL21, respectively. Cells were cultured (180 rpm) at 37 °C in LB broth with ampicillin (100 μg/mL), and the expression of recombinant proteins was induced by quickly cooling to 15 °C once the OD_600_ value reached 0.4–0.6, followed by further cultivation with isopropyl β-d-1-thiogalactopyranoside (0.1 mM) for 24 to 48 h. For Neg1-derivatives, cells were resuspended in PBS containing phenylmethylsulphonyl fluoride (0.2 mM) and dithiothreitol (1 mM), and sonicated (50-watt, 30 s) thrice on ice. The soluble fraction collected by centrifugation (10,000 rpm, 20 min, 4 °C) was applied to the column with TALON metal-affinity resin (Clontech Laboratories, Inc.), and washed with PBS. His_6_-tagged recombinant proteins were eluted with PBS containing imidazole (150 mM) and dialyzed against PBS (molecular weight cut-off: 3500 Da). SmBiT- or LgBiT-fused supBGRP was purified as previously reported [6]. The protein concentration was measured using a Pierce BCA protein assay kit (Thermo Fisher Scientific, IL, USA). Sodium dodecyl sulfate polyacrylamide gel electrophoresis (12% gel) was performed to confirm the expression of Neg1 and supBGRP derivatives.

### 4.6. Biotinylation of Neg1-E321Q

Purified Neg1-E321Q (400 μg/mL) was biotinylated by mixing with a five-fold molar excess of biotin-(AC_5_)_2_-*N*-hydroxysuccinimide ester (Dojindo, Kumamoto, Japan) in PBS at room temperature for 1 h and then stored at 4 °C until use [7].

### 4.7. Sandwich ELISA-Like Assay

A 96-well white plate (Greiner Bio-one, Frickenhausen, Germany) was coated with supBGRP-LgBiT (2 μg/mL) in PBS and incubated overnight at 4 °C. The plate was washed with PBS containing 0.05% Tween 20 (wash buffer) and blocked with 1% BSA-containing wash buffer (assay buffer) at room temperature for 1 h. After washing, the plate was incubated for 1 h with various soluble and insoluble glucan samples in assay buffer, and then washed with wash buffer. For the HRP-based ELISA-like assay, the plate coated with supBGRP-LgBiT was treated with Neg1-E321Q-biotin (2 μg/mL) for 1 h, washed, and further treated with streptavidin-HRP in assay buffer for 20 min. The peroxidase substrate, SuperSignal ELISA Femto (Thermo Fisher Scientific), was added after removing the unbound HRP. For the sandwich split-NanoLuc ELISA-like assay, plates incubated with various glucan samples were washed and treated with Neg1-E321Q-SmBiT (2 μg/mL) in assay buffer for 1 h. After washing, the bioactivity of the split-enzyme complex was monitored using the Nano-Glo luciferase assay reagent (Promega, WI, USA). Luminescence signals from HRP or NanoLuc were measured using a microplate reader (GloMax; Promega).

### 4.8. Split-NanoLuc Complementation Assay

A mixture solution (5 μL) of split-NanoLuc-fused glucan-binding probes (50, 100, 200, or 400 nM) was added to a two-fold serially diluted glucan samples (10 μL; seven concentrations from 1000 ng/mL to 50 μg/mL) in a 96-well white plate and incubated under shaking conditions (for 10, 30, or 60 min). The combination of supBGRP-LgBiT and Neg1-E321Q-SmBiT was used to analyze β-1,6-branched β-1,3-d-glucan, while the other combination of supBGRP-LgBiT and supBGRP-SmBiT was used to analyze the β-1,3-d-glucan structure. After shaking, 15 μL of furimazine solution (Nano-Glo luciferase assay reagent) was added to each well and the activity of reconstituted NanoLuc toward β-glucan was measured after 5–10 min using a GloMax luminometer with 1 s of integration time.

### 4.9. Preparation of C. albicans Cells

Yeast colonies on YPD agar were suspended in PBS and seeded at densities of 0, 10^4^, 10^5^, and 10^6^ cells/well in a 96-well white V-bottom plate (IWAKI, Shizuoka, Japan). Cells were then washed and resuspended in PBS (10 μL) for glucan analysis. In order to induce the hyphal form *C. albicans*, yeast suspended in culture medium comprised of RPMI 1640 medium (Thermo Fisher Scientific) and 10% heat-inactivated fetal bovine serum (Sigma-Aldrich) was cultured in 96-well white V-bottom plates (10^5^ yeasts/well) for 0–7 h at 37 °C. The plate was then washed twice with PBS to remove water-soluble molecules, and the hyphae resuspended in PBS (10 μL). In parallel, yeast (10^5^ yeasts/well) was cultured in the same conditions, in a 96-well clear flat-bottom plate (Sumitomo Bakelite, Tokyo, Japan) at 37 °C, and photographed using the EVOS FL Cell Imaging System (Thermo Fisher Scientific) at each incubation time (0–7 h) to evidence the growth of hyphae. The suspensions of yeasts or hyphae in the V-bottom plates were treated with a mixture of split-NanoLuc-fused glucan-binding probes (200 nM each, 5 μL) for 30 min. The furimazine solution (15 μL) was subsequently added, and NanoLuc activity was measured as described above.

### 4.10. Statistical Analyses

All statistical analyses were performed using GraphPad Prism 7.0 software (GraphPad Software, CA, USA). Based on the results of the Kruskal-Wallis test for normal distributions, significant differences were analyzed by two-tailed unpaired Student’s *t*-test or Welch’s *t*-test, or one-way analysis of variance (ANOVA) followed by multiple comparisons using Dunnett’s test or Tukey’s test.

## Figures and Tables

**Table 1:** Polysaccharides used in this study.

Sample	Structure	Source	Elemental Composition (%)		
			C	H	N
Zymosan A	β-1,6-/β-1,3-glucan	*Saccharomyces cerevisiae*	43.16	6.92	1.75
Curdlan	Linear β-1,3-glucan	*Alcaligenes faecalis*	39.67	6.75	0.03
Pustulan	β-1,6-glucan (slight β-1,3-glucan)	*Lasallia pustulata*	39.20	6.91	0.04
Scleroglucan	β-1,6-/β-1,3-glucan	*Sclerotium rolfsii*	40.42	6.66	0.84
Paramylon	Linear β-1,3-glucan	*Euglena gracilis*	41.88	6.59	0.00
Pachyman	β-1,6-/β-1,3-glucan	*Wolfiporia extensa*	40.24	6.82	0.08
Laminarin	mono-β-1,6-/β-1,3-glucan	*Laminaria digitata*	39.34	6.75	0.00
Yeast BG	β-1,6-/β-1,3-glucan	Yeast	42.99	6.93	1.83
Barley BG	β-1,3-/β-1,4-glucan	Barley	40.13	6.66	0.03
AgCAS	β-1,3-/β-1,6-glucan, others	*Agaricus brasiliensis*	-	-	-
HKCA	β-1,3-/β-1,6-glucan, others	*Candida albicans*	-	-	-
Chitin	β-1,4-poly-*N*-acetyl-d-glucosamine	Crab shell	-	-	-
Dextran	α-1,4-/α-1,6-glucan	*Leuconostoc mesenteroides*	-	-	-
Xylan	β-1,4-xylan backbone, others	Corn core	-	-	-
Mannan	α-1,6-/α-1,2-, α-1,3-mannan	*Saccharomyces cerevisiae*	-	-	-
Depleted zymosan	β-1,6-/β-1,3-glucan (insoluble)	*Saccharomyces cerevisiae*	-	-	-
CSBG	β-1,6-/β-1,3-glucan (soluble)	*Candida albicans*	-	-	-

-; not done.

**Table 2:** DNA fragments used in this study.

Primer/Fragment	Sequence
pColdI-n361-F	5’-TAGGTAATCTCTGCTTAAAAGCACAG-3’
NEG1-FS-R	5’-GCCGCCGCTGCCGCCGCCGCTGCCGCCCGCCCCTGCAGCCGG-3’
GS-SmBiT-SS	5’-GGCGGCAGCGGCGGCGTGACCGGCTACCGGCTGTTCGAGGAGATTCTGTAGGTAATCTCTGCT-3’
GS-SmBiT-AS	5’-AGCAGAGATTACCTACAGAATCTCCTCGAACAGCCGGTAGCCGGTCACGCCGCCGCTGCCGCC-3’

## Data Availability

Data is contained within the article.

## Abbreviations

BGRP	β-1,3-glucan recognition protein
BSA	bovine serum albumin
CSBG	Candida soluble β-glucan
ELISA	Enzyme-linked immunosorbent assay
GEM	glucan enzymatic method
HKCA	heat-killed *C. albicans*
HRP	horseradish peroxidase
LAL	*Limulus* amebocyte lysate
PBS	phosphate-buffered saline
